# American College of Medical Genetics and Genomics ACT Sheets Are a Vital Resource for State Newborn Screening Programs

**DOI:** 10.3390/ijns10040075

**Published:** 2024-11-19

**Authors:** Virginia Sack, Sara Etienne, Grace Beal, Sarah Bradley, Michele Caggana

**Affiliations:** 1 Newborn Screening Program, David Axelrod Institute, Wadsworth Center, New York State Department of Health, Albany, NY 12208, USA; grace.beal@health.ny.gov (G.B.); michele.caggana@health.ny.gov (M.C.); 2 Newborn Screening Program, Oregon State Public Health Laboratory, Portland, OR 97207, USA; sara.etienne@oha.oregon.gov

**Keywords:** newborn screening, public health, ACMG ACT sheets, genetic services

## Abstract

The American College of Medical Genetics and Genomics (ACMG) and the National Coordinating Center for the Regional Genetics Networks (NCC)-developed ACT sheets are a vital resource for state newborn screening (NBS) programs. They allow NBS programs to be able to provide up-to-date, just-in-time disorder information to primary care providers (PCPs). Their continued availability is necessary to ensure that all babies identified by newborn screening receive appropriate evaluation and care.

The ACT sheets, developed through a collaboration of the American College of Medical Genetics and Genomics (ACMG) and the National Coordinating Center for the Regional Genetics Networks (NCC), are an invaluable resource for state newborn screening (NBS) programs and the babies they aim to protect (https://www.acmg.net/ACMG/Medical-Genetics-Practice-Resources/ACT_Sheets_and_Algorithms.aspx (accessed on 6 November 2024)). Many NBS programs are chronically understaffed and underfunded, making the development of in-house educational materials an impossibility. Proper educational materials require not just an initial investment of resources to develop them properly, but also maintenance and updating over time as knowledge about a condition increases, technologies evolve, and new treatments emerge. The ACT sheets are regularly updated by clinicians with expertise in the featured disorders. These information sources, while not intended or appropriate for parents or the general public, are an ideal just-in-time resource for primary care providers (PCPs) when they are notified of an abnormal NBS result for one of their patients. We conducted a review of newborn screening program websites, which indicated that 25 out of 50 newborn screening programs have ACT sheets readily accessible to providers, and 2 other states have created their own provider fact sheets, but they contain links to, or are based on, the ACMG ACT sheets. Some states, like New Mexico, have partnered with the ACMG to create several individualized fact sheets for conditions, embedding state-specific support resources into the document.

In New York State, the Newborn Screening Program began sending the ACT sheets with all abnormal screening results in the Spring of 2020. During the early days of the COVID-19 pandemic, we heard from several of the clinical specialists who evaluate and perform workups on babies with positive newborn screens that they were being drafted to help on the front lines of their institutions’ pandemic response. Some institutions shifted to telehealth visits for outpatient appointments [1]. Prior to the pandemic, when babies had a positive newborn screen, the NBS program would inform their pediatrician as well as the closest specialist in that disorder, who would bring the baby in for evaluation. When the pandemic threatened this infrastructure, the NYS NBS program began sending PCPs the ACT sheets in addition to the abnormal screening results to ensure that these providers had readily available and reliable information regarding concerning signs/symptoms to watch for in these infants. The ACT sheets also provided PCPs with the recommended diagnostic laboratory tests needed to start the diagnostic evaluation for these babies. This was especially helpful in cases in which circumstances prevented a prompt visit with a specialist. Although the environment which prompted their use by the NYS NBS Program has subsided, the ACT sheets remain a staple for all abnormal result notifications to PCPs by the program. From 2021 to 2023, 5394 infants had a positive newborn screening result on the New York State newborn screening panel and required diagnostic testing and clinical evaluation, and 4543 of these referrals were for disorders with corresponding ACT sheets.

Oregon implemented the use of ACT sheets for all abnormal screening results in 2018, replacing their internally developed provider education materials. Due to diminished staffing and sparse program resources, the Oregon-developed sheets have become outdated; the last notable edits for all disorders were completed in 2004. Oregon’s program is a regional screening program. The ACT sheets are also used in Guam, Saipan, various military bases, and New Mexico to maintain consistency and equity throughout the region.

The ACT sheets serve to soften the effects of the gap in genetics coverage that becomes wider each year. There is a recognized shortage of geneticists in areas across the United States and throughout the world that will only increase as the current workforce ages. A 2005 report by Cooksey et al. [2] described the medical genetics situation as “critical”, and predicted it would become more serious in the future. Indeed, it has: a 2019 survey of US medical geneticists conducted by the ACMG through the NCC illustrated that there remains a gap between genetics services needed and workforce capacity [3]. The distribution of medical geneticists around the United States varies widely by state, with the highest concentrations seen in the Northeast and the mid-Atlantic, as well as in some mid-Western and Western states [4]. Twelve states had less than one medical geneticist, and Wyoming did not have any medical geneticists listed. Nationally, states averaged 2 medical geneticists per 500,000 people [4]. This barrier to accessing genetics services increases the disparity between different socioeconomic classes that universal NBS aims to eliminate. The ACT sheets empower PCPs to have the minimum information necessary to recognize concerning symptoms and initiate diagnostic testing in newborns with positive newborn screening results. While they may not provide a fix for the growing problem presented by the geneticist shortage, they do help to lessen the effect. For over 20 years, the ACMG, in its capacity as the NCC, received funding from the Health Resources and Services Administration (HRSA) to create ACT sheets, and more recently, to update them. Ongoing support is important for creating and maintaining the ACT sheets as they provide an invaluable resource to newborn screening programs and PCPs across the United States and around the world. The goal of newborn screening is to identify babies who may have serious medical conditions and ensure that they receive early workup, care, and treatment to optimize health outcomes. If babies are identified but are unable to receive timely care, then the system is not successful. The ACT sheets help to ensure that all babies identified by newborn screening can receive proper care, no matter what service barriers they may face; thus, it is critically important that this resource be maintained. Because new conditions under consideration for the federally recommended uniform screening panel are becoming increasingly complex, it is likewise important that new ACT sheets continue to be created to keep time with newborn screening expansion.

## Data Availability

No new data were created or analyzed in this study. Data sharing is not applicable to this article.
